# COVID-19 AND SOUTH AFRICA–CHINA ASYMMETRIC RELATIONS

**DOI:** 10.1177/00438200221102405

**Published:** 2022-06-10

**Authors:** Adeoye O. Akinola, Oluwaseun Tella

**Affiliations:** 61799 University of Johannesburg; 61799 University of Johannesburg

**Keywords:** BRICS, Coronavirus, COVID-19 Diplomacy, South Africa–China Relations, Africa, Africa–China Relations, South Africa’s Foreign Policy, Sustainable Development, Health Policy, Economic Policy, Ethnicity and Race, China’s Foreign Policy

## Abstract

While South Africa–China relations were only formalized in 1998, relations between these states date back to the 1800s. South Africa's quest for sustainable development through partnerships with global powers motivated its close ties with China. The 2015 Cape Town Declaration committed the two countries to improve health facilities and disease control. The coronavirus (COVID-19) pandemic presents an opportunity to rethink this partnership. Drawing on desktop research, this article engages the reality of COVID-19 and explores South Africa–China relations in the context of the pandemic. The emergence of the virus in China, its rapid spread, and the high fatality rate have had devastating repercussions across the world. This article argues that Beijing's response to COVID-19 raises more questions than it answers. The outbreak of the virus in China, its response, and emerging cases of racism and xenophobia against Africans in China also raise concerns about the future of South Africa–China relations.

## COVID-19 y las relaciones asimétricas entre Sudáfrica y China

Si bien las relaciones entre Sudáfrica y China solo se formalizaron en 1998, las relaciones entre estos países se remontan al siglo XIX. La búsqueda de Sudáfrica por el desarrollo sostenible a través de alianzas con potencias globales motivó sus estrechos vínculos con China. La Declaración de Ciudad del Cabo de 2015 comprometió a los dos países a mejorar las instalaciones de salud y el control de enfermedades. La pandemia del coronavirus (COVID-19) presenta una oportunidad para repensar esta asociación. Basándose en una investigación de escritorio, este artículo aborda la realidad de COVID-19 y explora las relaciones entre Sudáfrica y China en el contexto de la pandemia. La aparición del virus en China, su rápida propagación y la alta tasa de mortalidad han tenido repercusiones devastadoras en todo el mundo. Este artículo argumenta que la respuesta de Beijing al COVID-19 plantea más preguntas que respuestas. El brote del virus en China, su respuesta y los casos emergentes de racismo y xenofobia contra los africanos en China también generan preocupación sobre el futuro de las relaciones entre Sudáfrica y China.

## 2019 **冠状病毒病与南非-中国的不对称关系**

虽然南非-中国关系在1998 年才正式确立，但两国之间的关系可以追溯到1800 年代。南非通过与全球大国的伙伴关系寻求可持续发展，进而推动了其与中国的密切联系。2015 年，两国在《开普敦宣言》中承诺改善卫生设施和疾病控制。2019 冠状病毒病（COVID-19）大流行为重新思考该伙伴关系提供了机遇。基于桌面研究，本文在大流行情境下研究COVID-19 的现实，并探究南非-中国关系。该病毒在中国的出现、迅速传播和高致死率已在世界范围内产生了毁灭性的影响。本文论证认为，北京方面的 COVID-19 响应所引发的问题多于其给出的答复。该病毒在中国的爆发、其响应措施以及针对在华非洲人的种族主义和仇外心理的新兴案例也引发了对未来南非-中国关系的担忧。

South Africa became a democratic state in 1994, ending its international isolation. The new government immediately set about reviewing its foreign policy and reaching out to the international community (Grimm et al. 2013). Like other African states, South Africa's quest for sustainable economic development through partnerships with global powers motivated its close ties with China. Beyond economic considerations, Pretoria has collaborated with Beijing in the area of health. Following the outbreak of COVID-19 in China and its spread to other countries, Beijing reiterated its commitment to support the fight against the deadly pandemic in Africa and South Africa in particular. In recent years, the South African state has shown relatively weak capacity in terms of service delivery including in the health sector. Thus, the country needed China and other global powers’ assistance to tame the pandemic.

While the South African economy has fared relatively well in comparison with other African countries, the country has not been able to fulfil social needs, particularly in terms of reducing structural violence, creating employment, and improving the wellbeing of its citizens (Wöcke and Sing 2013). Its relations with China promised to accelerate the country's development post-apartheid (Monyae and Band 2018). In general, African states are wary of the West due to its colonial history of exploitation. In a bid to curtail the overwhelming influence of Western powers and promote “Southernization,” South Africa has collaborated with powerful developing countries on debt relief, reform of multilateral institutions such as the International Monetary Fund (IMF) and global free trade (Marthoz 2012, 3). It is within this context that one can understand Pretoria's membership of the group of emerging economies, Brazil, Russia, India, China, and South Africa (BRICS) and the moribund forum comprising India, Brazil, and South Africa (IBSA).

South Africa–China relations transcend the conventional resource extraction and infrastructure financing that define China's ties with other African countries. South Africa is home to the largest and oldest Chinese community in Africa, hosts a number of Confucius Institutes at its universities, including Johannesburg, Stellenbosch, and Rhodes, and accommodates an active Chinese media sector. For example, in 2013, two Chinese state-backed companies bought a 20 percent stake in Independent Media, South Africa's second largest media company (City Press 2020). Chinese firms have also been involved in media production and distribution in South Africa, including content production, distribution, development of infrastructure, and the training of journalists (Madrid-Morales and Wasserman 2017).

China and South Africa have collaborated to forge a common global agenda through the BRICS, the United Nations (UN), and the Group of 20 (G20). Two key examples are their rejection of the West's criticism of the January 2019 presidential elections in the Democratic Republic of Congo (DRC) that resulted in the emergence of Félix Tshisekedi as president, and their opposition to the U.S.-led “regime change” agenda in Venezuela in the same year. However, the two countries have also displayed contrasting interests, especially in complex regional security issues such as the violent Sudan–South Sudan conflict and the Zimbabwean political impasse during Robert Mugabe's regime. This reveals the complexity in South Africa–China relations. Aside from international pessimism toward South Africa–China relations, many Africans are skeptical about China's rising influence on the continent. Beijing's “coronavirus diplomacy” is seen “as part of a broader strategy to help it deflate some of the blame” (Asiedu 2020). Indeed, many African countries are suspicious of China's quest to support the fight against COVID-19, evident in the continent's ambivalence toward Chinese vaccines including Sinovac and Sinopharm.

Nevertheless, South Africa continues to enhance Africa–China ties and Pretoria has become the reference point to evaluate China's relations with the continent. In what Alden and Schoeman (2015) describe as “symbolic representivity,” South Africa's membership of international organizations such as the G20 and BRICS accounts for the country's status and moniker as a regional and middle power (Marthoz 2012).

In 2019—following President Xi Jinping's declaration of China's strategies as a global power at the official opening of the 19th National Congress of the Chinese Communist Party in 2017—the Chinese president asserted that “no force can shake the status of this great nation” (Tella 2020, para. 1). Similarly, former Chinese Ambassador to South Africa, Lin Songtian, changed the narrative of global economic dominance and declared China as the global economic giant (du Plessis 2020). Despite the rise of China in past decades, Tella (2020) asserts that the outbreak of COVID-19 and Beijing's initial response have dented its quest for global superpowerhood (see also Tella 2016). In the four decades prior to the emergence of COVID-19, China's Gross Domestic Product (GDP) grew at an average of 9.5 percent a year, against a global average of 2.9 percent (Ming 2019). Lin noted that China had accounted for 30 percent of global growth since 2008 and declared that “If China gets a cold, everyone will sneeze” (du Plessis 2020).

The emergence of COVID-19 in China resulted in states across the world adopting and enforcing measures, including lockdowns, to contain its spread. By April 2020, the Chinese government claimed to have rid the country of the disease, relaxed restrictions on people's movement, and gradually returned to normalcy. However, the resurgence of the pandemic in cities such as Wuhan, Nanjing, and Shanghai has resulted in a return to fairly stringent measures. Following COVID-19's spread to South Africa and as a part of the South Africa–China partnership, the Chinese Consul-General to South Africa commended Pretoria's efforts to combat the virus and pledged Beijing's support. It is against this backdrop that this article draws on desktop research to present the reality of COVID-19 and explore South Africa–China relations in the context of the pandemic. The article comprises six sections. We first present an introductory overview and highlights the article's objectives before examining the evolution of South Africa–China relations. We then engage the contradictions inherent in these bilateral relations. Next we explore the nature of the COVID-19 pandemic and engage the reality of COVID-19 and South Africa–China relations. The final section offers some conclusions.

## The Evolution of South Africa–China Relations

South Africa–China engagement should be understood within the broad conversation on China–Africa relations as the latter dominated public discourse in 2006 after more than 40 African heads of state attended the triennial Forum on China–Africa Cooperation (FOCAC) in Beijing (Bodomo 2009). The forum is the largest assembly of African leaders outside UN meetings. Aside from the rise in Chinese investment in African countries like Nigeria, Egypt, Ethiopia, and South Africa, the Chinese migrant community in Africa has increased considerably, particularly in South Africa where it exceeds 350,000, the largest on the continent (Monyae and Band 2018). South Africa receives an average of 100,000 Chinese tourists per annum, contributing to the growth of tourism and the country's GDP (Government of South Africa 2020a). In recognition of China as an important tourism market, the Department of Tourism started training local tourist guides in Mandarin for effective communication with Chinese tourists.

China invested heavily in the African Union (AU), and member states reciprocated by supporting China on the global stage. For instance, the bloc vote by African representatives at the International Olympic Committee (IOC) earned China the right to host the Beijing Olympics (Bodomo 2009). Similarly, in the UN, African states have opposed the West's efforts to formally condemn China's human rights record (Hanauer and Morris 2014). Data released by China's Ministry of Foreign Affairs reveals that:

Up to now, Chinese investment and financial support in Africa have exceeded US$110 billion in accumulative terms. So far, upholding the idea of intensive development, China has financed and built more than 6,000 km of railway, over 10,000 km of roads, over 80 power plants, more than 200 schools, over 60 airports, ports, and bridges, as well as dozens of industrial parks and special economic zones. It has strongly facilitated Africa's industrialization, urbanization, and self-sustainable development (MFAPRC 2019).

Such investment in the infrastructural development of many African countries like Nigeria and Ethiopia reinforces optimism with regard to South Africa–China ties. According to Monyae and Band (2018), the South African socio-political elite sees the state's relations with China as a key condition for achieving its strategic development goals. In an interview, Lin noted that South Africa was the first African country to sign a Memorandum of Understanding (MOU) on the Belt and Road Initiative (BRI) Cooperation with China (MFAPRC 2019). Thirty-nine African countries and the AU have since climbed on this bandwagon. Many would agree with Marthoz (2012, 2) that, since the demise of apartheid, “South Africa is undoubtedly the most powerful African nation” within the international system. While Nigeria's GDP exceeds that of South Africa, the latter is the most industrialized and advanced economy on the continent (Ogunnubi and Akinola 2017).

South Africa's engagement with China rests on the following four foundations:

China's contribution to the defeat of colonization in the rest of Africa, and particularly apartheid in South Africa; China and South Africa's shared strategic approach to global issues and international relations; China's support of Africa's development efforts, coinciding with South Africa's foreign policy commitment to the African agenda; and the Brazil, Russia, India, China, South Africa alliance (BRICS). (April and Shelton 2014, 1)

China spearheaded South Africa's admission to BRIC in 2010 and the name changed from BRIC to BRICS. The acronym BRIC was coined by Jim O’Neill in 2001 to represent the association of four countries (Brazil, Russia, India, and China) that possessed the “counterweight to global imperialism.” In 2010, BRICS expanded its scope beyond economic issues to accommodate diplomatic and socio-political concerns. China and South Africa aim to present a united position in the UN and FOCAC. While South Africa is optimistic that it will receive China's support in its quest to reform the UN and to accommodate South Africa as a permanent member of a reformed United Nations Security Council (UNSC), China has not shown much enthusiasm in expanding membership of the UNSC as this could invigorate India and Japan's—states in its primary sphere of influence—quest for permanent membership.

Chinese officials have tagged South Africa as a “locomotive” for Africa's industrialization and regard the country as the gateway to the continent (Yu-Shan 2017). However, rather than acting as the voice of Africa through its membership of key global organizations, “South Africa seems to have used its influence solely to expand international trade for itself rather than genuinely seeking regional trade” (Ogunnubi and Akinola 2017, 6). South Africa continues to play a significant role in the African political economy through the presence of companies like MTN, Vodacom, Multi Choice, and Game in many African states. More than 120 and around 230 South African companies have set up branches in Nigeria and Tanzania, respectively (Tella 2021). In this manner, Chinese industrial byproducts—which are the mainstay of these companies—are transferred across Africa.

Relations between the Chinese and South Africans predate the apartheid and post-apartheid dispensations. The relations can be traced to the nineteenth century when Chinese laborers migrated and settled in South Africa to work in the gold mines. “Chinese diplomatic representation was established in the early 1900s to support these laborers. Between 1904 and 1910 there were almost 64,000 Chinese working on the Witwatersrand gold mines near Johannesburg” (Burke, Naidu, and Nepgen 2008, 5). Following the emergence of the National Party Afrikaner government in 1948, like others termed “non-whites” by the regime, the Chinese community suffered racial discrimination (Alden and Wu 2016). Although China supported the struggle against apartheid from the 1950s, South Africa's international isolation dictated its quest to find friends and by 1980, the apartheid regime had established military cooperation and economic ties with China (Alden and Wu 2016). However, formal diplomatic ties were only established in 1998 (Alden and Wu 2016; Grimm et al. 2013). The relations were initiated by Beijing, Gauteng, Shandong, and the Western Cape, which established sister-city and sister-province partnerships (Grimm et al. 2013).

Following the establishment of diplomatic relations in 1998, the formation of the South Africa–China Bi-National Commission in 2000 and the elevation of relations to a comprehensive strategic partnership in 2010, Pretoria-Beijing relations have arguably blossomed. They have since been anchored on four key initiatives: FOCAC, BRICS, the BRI, and South–South cooperation (Grobbler 2020) and are reinforced by an increasing number of people-to-people exchanges. The Bi-National Commission sought to create enabling environments on both sides for expanded economic activity, especially in mining and manufacturing. Celebrating the 20th anniversary of the supposedly symbiotic relations in 2018, Presidents Cyril Ramaphosa and Xi Jinping underlined the salience of the comprehensive strategic partnership, highlighting political trust, strategic cooperation, economic cooperation, and people-to-people exchanges (Grobbler 2020).

The diplomatic relations are further guided by principles such as development, mutually beneficial collaboration, and non-interference in each other's local politics (April and Shelton 2014, 3). For instance, China has stayed out of the land expropriation without compensation discourse. Other global actors like the United Kingdom, Australia, and the United States have expressed keen interest in the fate of South African minority white farmers and landowners who may be the ultimate losers in the government's proposed policy (Akinola 2019). South Africa has reciprocated by refraining from opposing Beijing's internal politics and foreign policy behavior.

Indeed, South Africa–China relations reflect post-apartheid South Africa's recalibration of its foreign policy from the erstwhile Americentric and Eurocentric humanistic values to developmental pragmatism. This is evident in its foreign policy behavior while serving three terms as a non-permanent member of the UNSC and its stance on the Dalai Lama (Alden and Wu 2016). However, the relations are more complex than they seem as there are domestic concerns around Chinese firms’ competition with local companies and the impact of the relations on South Africa's idealistic foreign policy objectives of promotion of democracy, human rights, and a just international legal order.

Pretoria and Beijing have collaborated in key strategic areas. For example, China identified skills shortages as a decisive factor militating against the development of South African economy. It announced that about US$6.3 million would be allocated for technical training in South Africa in 2006, and this was extended by an additional US$25 million in February 2007 (Burke, Naidu, and Nepgen 2008). Beijing's interest in South Africa is driven by the latter's “vast range of mineral resources, chemicals, pulp and paper, textiles, clothing, live animals, [and] agricultural products” (Owusu-Sekyere 2017, 12). The Industrial and Commercial Bank of China (ICBC) assumed a 20 percent stake ($5.5 billion) in South Africa's Standard Bank, one of the largest foreign direct investments in the country. In similar fashion, South Africa's company Naspers made inroads into China's largest Internet company, Tencent (Alden and Wu 2016). The countries’ two-way trade accounted for nearly 1/4 of China's total trade with Africa. Chinese investment and financing in South Africa exceeded US$25 billion. All the major Chinese financial institutions have set up branches in Johannesburg. China and South Africa have agreed on reciprocal arrangements of international settlement in local currency and Chinese RMB has become the reserve currency of South Africa (MFAPRC 2019).

The ties between China and South Africa are not only entrenched by the volume of trade and investment, but by other avenues such as the movement of people in diverse exchange programs and cultural diplomacy. Providing a comprehensive perspective on relations between the two countries, the Parliamentary Monitoring Group (PMG) states that “The Pretoria Declaration on Partnership was issued in April 2000. A Bi-National Commission was established in 2001. A Strategic Partnership was declared in 2004. A Program for Deepening Strategic Partnership was established in June 2006. A Comprehensive Strategic Partnership was agreed upon in principle during 2010” (cited in Grimm et al. 2013, 13).

Minerals are exported from South Africa to boost China's industrial productivity, including material to manufacture mobile phones, which are exported to South Africa as finished goods. A report showed that the number of mobile phones imported from China exceeds South Africa's population (April and Shelton 2014, 1). South Africa telecommunications company Cell C and the China Development Bank concluded a loan deal of $303.6 million, while Chinese First Automobile Works invested $100 million in the construction of an automobile assembly plant in the Eastern Cape, South Africa, creating around 1,000 jobs (April and Shelton 2014, 2). The Industrial and Commercial Bank of China (ICBC) invested $4.46 billion in Standard Bank of South Africa, China's largest financial investment in 2007 (April and Shelton 2014). China's investment in South Africa began to escalate in 2009. While the value of such trade was estimated at R121 billion in 2008, it rose to R205 billion in 2012 and surged to R270 billion in 2013 (Alden and Wu 2016; Monyae and Band 2018). By 2017, China's trade with South Africa was estimated at $39 billion (R455bn) (Yu-Shan 2017).

## South Africa–China Relations: Should South Africa Be Worried?

In the initial stage of South Africa–China relations, more South African companies penetrated China than Chinese firms advanced into South African markets (Bodomo 2009). Naspers, SASOL, AngloCoal, Spur, Standard Bank, and Old Mutual are active players in Chinese domestic markets. However, over the years, China has significantly increased its presence in South Africa. Unlike China, which provides assistance and resources to its companies operating abroad, the “South African government does not appear to provide any significant support to South African corporations operating in China beyond consular assistance” (Burke, Naidu, and Nepgen 2008, 13).

Between 2003 and 2017, Chinese companies invested around $15.2 billion in the form of stock FDI in South Africa and in 2017 alone, trade between South Africa and China was worth $39.17 billion, representing about 30 percent of the former's total trade (Gouvea, Kapelianis, and Li 2020). By mid-2021, the China–Africa Development Fund had committed to invest more than $5.5 billion in 37 African states. Coupled with Chinese companies’ $26 billion investment and financing across Africa, cumulative investment exceeded $25 billion (Grobbler 2021). The development fund has invested and financed major projects in key sectors in South Africa including electricity, energy, culture, cement, and home appliances (Grobbler 2021). This has not proven difficult as China has been South Africa's largest trading partner for more than a decade. Indeed, more than 180 Chinese multinationals and thousands of small- and medium-sized enterprises (SMEs) have established a footprint in South Africa, partly due to the fact that the country is the major African actor in renewable and sustainable energy, which is critical for Chinese energy companies (Gouvea, Kapelianis, and Li 2020). Given that Chinese state-owned companies such as Longyuan Power and United Power and private enterprises like BYD Company Limited are key actors in the South African energy industry, it is not surprising that the development of green energy in the country was one of the major topics at the 2015 FOCAC summit. Other key Chinese companies in South Africa include FAW in the manufacturing sector; the Construction Bank of China, Bank of China (BOC), China Development Bank and Industrial and Commercial Bank of China (ICBC) in the banking industry; HiSense in the electronics sector; the Jinchuan Group and China Investment Corporation (CIC) in the mining sector; Beijing Automobile International Corporation (BAIC) in the automobile sector; and the Ji Dong Development Group in the cement industry (Gouvea, Kapelianis, and Li 2020). A few South African companies operate in China, including the Naspers media company and South African Breweries’ (SABMiller) joint ventures in the country (Gouvea, Kapelianis, and Li 2020). Tables 1, 2, and 3 reveal the extent of trade relations between the two states.

**Table 1.** China's Largest Export Markets in Africa (2019).

Country	Exports (in $ Billion)	Percentage of Chinese Exports to Africa
South Africa	16.6	14.6
Nigeria	16.6	14.6
Egypt	12.2	10.7
Algeria	6.9	6.1
Kenya	4.9	4.3
Ghana	4.9	4.3
Total Six Countries		54.6

*Source:*Stein and Uddhammar (2021).

**Table 2.** China's Key Import Destinations in Africa (2019).

Country	Imports (in $ Billion)	Percentage of Chinese Imports from Africa
Angola	23.3	29.8
South Africa	9.5	12.1
Republic of Congo	5.9	7.5
DRC	4.9	6.2
Libya	4.7	6.0
Gabon	4.6	5.9
Total Six Countries		67.5

*Source:*Stein and Uddhammar (2021).

**Table 3.** Main Recipients of Chinese FDI (2019).

Country	Total Stock (in $ Billion)	Percentage of Chinese FDI in Africa
South Africa	6.1	13.8
DRC	5.5	12.5
Angola	2.9	6.5
Zambia	2.8	6.5
Ethiopia	2.5	5.6
Ghana	1.8	4.1
Total Six Countries	21.6	49.1

*Source:*Stein and Uddhammar (2021).

One of the contradictions of these trade relations is South Africa's trade deficit with China. This is due to South Africa's “high imports of value-added goods and China's increasing demand for mineral products,” which may hamper South Africa's industrial growth (Alden and Wu 2016). South Africa has “removed most of its import duties on Chinese goods, but China continues to maintain import duties on South African agricultural goods, resulting in a 7.17 percent duty asymmetry in favor of China” (Monyae and Band 2018, 6). Many hold that “China is sapping Africa's manufacturing potential and also extracting Africa's resources without any significant benefits to Africa. Thus, China is seen as a contributor to Africa's underdevelopment and deindustrialization” (Mlambo, Kushamba, and Simawo 2016, 257). China's investment in its manufacturing sector and export of finished goods have led to low levels of skills and technology transfer and has not enhanced local job creation (Owusu-Sekyere 2017). South Africa recorded a trade surplus of R846.6 million in vegetable exports to the United States but suffered a deficit of R6 billion with China between 2010 and 2016 (Owusu-Sekyere 2017). China's “fraudulent act of exporting dumping products” (Tshedza and Ephraim 2021, 122) is further proof of the asymmetrical relations between these states. For example, in 2014, Chinese and South African customs data on garments differed significantly. While China's reports claimed that the global power had exported garments to the value of US$1.71 billion, South Africa's data reflected US$945 million (Tshedza and Ephraim 2021). This demonstrates the unfair trade regime that has characterized China's image in the global arena.

Marthoz (2012, 4) notes that South Africa's leading trade union federation, the Congress of South African Trade Unions (COSATU) believes that China poses a threat to South Africa's economic interests and “even evokes the specter of a new era of colonialism.” For example, local textile and garment industries have suffered due to the import of cheap, mass-produced Chinese products that have dominated South African markets (Grimm et al. 2013). The authors add that China's exports to South Africa have led to the loss of about 75,000 jobs in the manufacturing sector. While these realities and the asymmetrical nature of the relations have triggered negative perceptions of China's economic interests in South Africa, they have afforded poor households the opportunity to purchase cheaper goods.

Aside from asymmetric trade relations, China's treatment of South Africans leaves much to be desired. In 2015, the Chinese government arrested 20 foreigners, including ten South Africans “without charge” (Bernardo 2015). Of these, 11 were released, leaving five South Africans, three Britons, and an Indian national. The Chinese authorities accused some of them of watching propaganda videos from a banned armed group in their hotel rooms. The arrests generated mixed reactions across South Africa, with Pretoria disappointed in light of the seemingly strong ties between the states. While it was “inconceivable” that this happened when China played host to the South African President, Pretoria subsequently utilized its diplomatic relations with China to secure the release of the South African detainees (Mail and Guardian 2015). In 2018, the Department of International Relations and Cooperation (DIRCO) raised the alarm over the recurring arrest and detention of young South Africans in foreign countries, particularly in China. During this period, about 98 South Africans were held by Beijing for visa-related offenses (Grobler 2018). Similar maltreatment and discrimination against Africans have been reported in China during the COVID-19 pandemic. DIRCO stated that

South Africa is deeply concerned about reports of alleged ill-treatment of African nationals in China, including the forceful testing, quarantining for COVID-19, and other inhuman treatment. Reports of these alleged actions are inconsistent with the excellent relations that exist between China and Africa, dating back to China's support during the decolonisation struggle in Africa, and now manifesting in an extensive Africa-China partnership. This collaborative, mutual relationship also finds concrete expression in the support that China is providing to Africa in the ongoing fight against the COVID-19 pandemic. South Africa welcomes the action taken by the AU Commission Chairperson, Mr. Moussa Faki Mahamat, to summon the Chinese Ambassador in Addis Ababa to provide an explanation and express the AU's deep concern about this matter. (DIRCO 2020)

It is against the backdrop of this criticism coupled with Africa-wide backlash that the Chinese government deployed public diplomacy to downplay the incident, stressing that Beijing does not pursue an Afrophobic foreign policy. However, prior to the emergence of COVID-19, Africans living in China were associated with negative stereotypes such as drug trafficking and other crimes (Kohnert 2022) that are perpetuated by the Chinese media and the police. For example, in 2016, a Chinese laundry detergent commercial depicted a black man turning into a light-skinned Chinese man after he had been put in a washing machine (Kohnert 2022). The Chinese police are notorious for racial profiling of African migrants who are easy prey in their quest to achieve their performance targets. An example is Public Security Bureau in Guangzhou's categorization of Africans as “triple illegal” (Kohnert 2022). These perceptions have been reinforced by the pandemic, with discrimination and xenophobic attacks orchestrated against African migrants.

Another important example of Beijing's exercise of somewhat hegemonic influence on South Africa is the latter's denial of a visa to Tibetan leader the Dalai Lama three times within the space of five years including in 2009 when his visa application to attend a peace conference with Nelson Mandela, Desmond Tutu, and FW de Klerk, his fellow Nobel Peace Laureates was denied (Jacobs and Yu 2014). He was also denied a visa to attend Tutu's 80th birthday in 2011 and in 2014 to attend the 14th World Peace Summit (Tella 2018). This is attributed to Beijing exerting pressure on Pretoria and the possibility of economic consequences should South Africa not heed the wishes of its largest trade partner. Given China's violation of human rights in Tibet, Pretoria's international reputation as a defender of such rights was severely dented. China also used South Africa as a major route to transport weapons to Zimbabwe during Robert Mugabe's regime. In 2018, Pretoria turned a blind eye to a Chinese ship carrying 77 tons of arms to Zimbabwe, including around three million rounds of ammunition, 2,500 mortar rounds, and 1,500 rocket grenades (Kinyondo 2019). Given the human rights abuses that characterized the Mugabe era, Pretoria was again arguably arm-twisted by Beijing despite the fact that the repressive regime might use the arms to crush its opposition.

## Understanding the COVID-19 Pandemic

COVID-19 was first discovered in a 55-year-old Chinese citizen in Hubei province on November 17, 2019, long before the Chinese government acknowledged and announced the emergence of the deadly virus. By the end of 2019, 266 Chinese people had contracted the disease (The Guardian 2020). On January 8, 2020, the virus was declared the Novel Coronavirus and, two days later, China shared the health genetics of COVID-19 with the World Health Organization (WHO). The Chinese government announced human-to-human transmission of the virus on January 21, 2020. With a population of 1.4 million, the country responded by mobilizing more than 41,000 health practitioners and building two sophisticated hospitals within two weeks to combat the spread of the virus (Government of South Africa 2000a). By February 28, 2020, there were 77,780 confirmed cases of COVID-19 in China, with 27,377 people having recovered and been discharged from hospitals, and 2,666 deaths (Government of South Africa 2000a).

China's domestic response to the spread of the disease is laudable. By September 2, 2021, the country had recorded 123,103 confirmed cases and 5,684 deaths, and 2,040,871,973 vaccine doses had been administered (WHO 2021). However, its response at the global level has arguably been less successful and the fight against the pandemic has been led by the West. Vaccines like Pfizer, Oxford/AstraZeneca, and Johnson & Johnson produced in Western countries have been globally accepted, while skepticism persists in relation to Chinese vaccines such as Sinovac and Sinopharm. Thus, China's initial diplomatic gains due to its aid and medical assistance to states around the world have been overshadowed by the West's triumph in vaccine diplomacy.

China is noted for regulating the media in a bid to suppress any information that reveals Beijing's weaknesses. There is compelling evidence that it continues to hide certain information concerning the outbreak of the virus, particularly in the initial stage of its discovery. This jeopardizes not only the wellbeing and survival of the Chinese, but the entire world. The government allegedly detained the “brave doctors and whistleblowers who attempted to sound the alarm and warn their fellow citizens when they understood the gravity of what was to come” (Hamid 2020, para. 1). The general feeling was that, while China acted decisively against the doctors and whistleblowers, it was slow in its response to the destructive virus (Guardian 2020). Both China and the world were to pay dearly for this. By the time concerted efforts were made to contain the virus in Wuhan—the city where it originated—more than five million people had already left the city (Hamid 2020).

Controversy surrounds debates on the origins of the coronavirus, but scientists agree it is caused by the SARS-CoV-2 virus (Hassanin 2020). At the initial outbreak, they held that it may have developed in bats, and later pangolins, but “genomic comparisons suggest that the SARS-Cov-2 virus is the result of a recombination between two different viruses, meaning the exact origin of the virus is still unclear” (Hassanin 2020). Research has found that COVID-19 is associated with SARS-CoV, which was “responsible for an epidemic of acute pneumonia which appeared in November 2002 in the Chinese province of Guangdong and then spread to 29 countries in 2003.” China recorded 8,098 cases, including 774 deaths (Hassanin 2020). The disease quickly spread across the world.

As at March 19, 2020, global infections stood at 209,839 (16,556 new cases), with 8,778 deaths (828 new deaths) (WHO 2020a). The figures increased sharply, and by March 30, 2020, there were 693,224 confirmed cases (58,411 new cases), and 33,106 deaths (3,215 new deaths) (WHO 2020b). Table 4 shows the rate of infection and deaths in different regions in the initial stages.

**Table 4.** Data at the Peak of COVID-19 between March 19 and 30, 2020.

	Continent	Number of Cases/New cases		Number of Deaths/New Deaths	
S/N	Period	19 March	30 March	19 March	30 March
1	Western Pacific Region	92,333 (488)	103,775 (987)	3,377 (20)	3,649 (23)
2	European Region	87,108 (10,221)	392,757 (31,726)	4,084 (591)	23,962 (2,535)
3	Southeast Asian Region	657 (119)	4,084 (375)	23 (14)	158 (19)
4	Eastern Mediterranean Region	19,518 (1,430)	46,329 (3,552)	1,161 (150)	2,813 (145)
5	Region of the Americas	9,144 (4,166)	142,081 (21,289)	119 (50)	2,457 (484)
6	African Region	367 (132)	3,486 (482)	7 (3)	60 (09)

*Source:*World Health Organization (2020a, 2020b).

The high rate of infection and deaths within the 11-day period reflected in the above table was a source of major concern to the global community—of even greater concern was the recording of asymptomatic transmission. Hartley, Reisinger, and Perencevich (2020, 1) note that

Increasing evidence suggests that the time from infection to infectiousness (the latency period) is less than the time to symptoms (the incubation period). Thus, people may inadvertently infect others before realizing they are infected because they are not yet experiencing symptoms. Asymptotic cases may be particularly common in children and young adults.

The pandemic has since wreaked havoc, although there has been relative success in the global fight to contain it, especially through the development and mass production of vaccines. By September 2, 2021, there were 218,205,951 confirmed cases and 4,526,583 deaths, and 5,289,724 918 vaccine doses had been administered (WHO 2021). By May 5, 2022, there were 513,955,910 confirmed cases of COVID-19, including 6,249,700 deaths, reported to WHO and a total of 11,562,157,794 vaccine doses had been administered (WHO 2022).

“The crude case fatality ratio is estimated to be significantly higher than seasonal influenza and is dramatically higher in the aged and those with underlying co-morbid conditions (especially cardiovascular disease, pulmonary disease, and diabetes) regardless of age” (Hartley, Reisinger, and Perencevich 2020, 2). Due to the initial lack of drugs and/or vaccines, the authors warned against “waiting until community transmission is detected” (Hartley, Reisinger, and Perencevich 2020, 2) before acting through social distancing, restriction of movement, and personal hygiene. Countries such as South Africa have devised many strategies, including the aforementioned, to contain the spread of the disease. Furthermore, the country's management of the virus and Beijing's response have revealed the reality of South Africa–China partnerships.

China has been a major actor in the containment of similar diseases in Africa. Beijing collaborated with African countries and the international community to contain the Ebola epidemic that erupted in West Africa. The Chinese government provided technical and non-technical assistance to 13 affected African countries—such as Guinea, Liberia, and Sierra Leone—estimated at around US$120 million, donated Ebola protection kits and mobile laboratory testing automobiles and constructed national Ebola research facilities (Tambo et al. 2016). In 2015, the Second Ministerial Forum of China–Africa Health Development, which was held in South Africa (Cape Town) adopted the Cape Town Declaration and highlighted the implementation framework to promote China–Africa collaboration in public health. In terms of the declaration, China subsequently pledged US$60 billion to address major health-related challenges and issues in Africa (Tambo et al. 2016).

After the outbreak of COVID-19 in South Africa on March 6, 2020, with a single case in KwaZulu-Natal, the Chinese Ambassador to the country, Lin Songtian reiterated his country's confidence in South Africa's health system and China's support to combat the disease, “We speak very highly of our relationship. We are partners, not only in good time, but also in bad time. We are fighting together, we are ready. We already maintain close contact with your (South Africa's) government, your ministry of health and other departments in the government” (du Plessis 2020). By March 5, 2020, new confirmed cases of the virus in China had dropped to an average of 143 per day from 3,887 per day in early February. The average daily number of deaths was 30, with a death rate of 3.7 percent and a recovery rate of about 66.7 percent (Du Plessis 2020). Within a month, the figures in South Africa skyrocketed. By April 9, the country had recorded more than 1,800 cases, the most in Africa. Data released by the Minister of Health, Zweli Mkhize less than a week later (April 14, 2020) recorded 2,506 cases and 34 deaths (Meyer 2020).

By July 2021, Pretoria had approved the Chinese vaccine Sinovac for local use. Given the increasing number of confirmed cases, which reached 2,777,659 total cases and 82,261 deaths, by September 2, 2021 (WHO 2021), Pretoria arguably made a desperate bid to save lives and reduce the burden on its health facilities.

## The Reality of COVID-19 and South Africa–China Relations

In the initial stage of the pandemic, South Africa commended China for the manner in which it managed the disease. However, as events unfolded, Pretoria resorted to silence on China's response. It is possible that the South African government did not initially have a full grasp of the depth of the issues and the virus’ impact on the South African community in Wuhan and its surrounding areas. This diplomatic naivety was partly due to Beijing's hoarding of information and suppression of the reality. Due to pressure from affected South Africans in China and their local sympathizers, the government was confronted with the moral imperative to repatriate them. The South African government declared that repatriating 184 South Africans (the majority of whom were students and teachers) living in Wuhan would cost taxpayers R25 million (Young 2020). Ultimately, 114 South Africans were repatriated on March 14 from Wuhan to Limpopo (the quarantine site) in what was a mixture of “concern and anger” (Young 2020).

Some South African citizens were critical of this decision due to “the cost of expatriation and the potential health risk of bringing citizens from [a] highly infected region into the country” (Young 2020). However, the government ignored the few opposing voices, including that of Lin Songtian, to promote its national interests by repatriating its citizens from China. Irrespective of bilateral ties, national interests are key to inter-state relations in the international system. The identification of a national interest determines the direction of a nation's foreign policy and diplomatic relations. South Africa is a constitutional democracy and its 1996 constitution remains the bedrock of its relations. The constitution mandates the government to protect South Africans’ “right to life,” under Section 11 of the Bill of Rights (Department of Justice 1996).

Apart from protecting the lives of South Africans in China, the government also needed to act promptly to preserve lives and contain the spread of the virus within its territory. Its strategy included the declaration of a state of disaster and subsequent national lockdown from March 26, 2020. The government also announced an intensified public health response to slow down and reduce infections, a comprehensive package of economic support measures to assist businesses and individuals affected by the pandemic and increased social support to protect poor and vulnerable households. Before the completion of the hard lockdown, which was to end on April 16, 2020, the presidency announced its extension. Justifying the extension of the lockdown from April 16 to May 1, 2020, President Cyril Ramaphosa noted, “if we end the lockdown too soon or too abruptly, we risk a massive and uncontrollable resurgence of the disease. We risk reversing the gains we have made over the last few weeks, and rendering meaningless the great sacrifices we have all made” (The Presidency 2020). The rate of infection and deaths fell short of government's projections, which is attributable to the decisive action taken to shut down the country.

Beijing's standpoint on the outbreak of COVID-19 in South Africa was relayed through Lin Songtian who asserted that evacuating foreign nationals from China would worsen the global spread of the infection (Fabricius 2020). China was also uncomfortable with Pretoria's decision to place an embargo on flights from China and seven other highly infected countries. Beijing recalled Lin Songtian in the wake of the spread of the virus and the quest to expatriate South African citizens from the Wuhan area. Lin was no ordinary ambassador and his profile in Beijing and Africa is noteworthy. Prior to his appointment in 2017, he held ambassadorial positions in Liberia, Malawi, and Zambia and acted as Director General for China–Africa relations, making him one of the major architects of China's engagement with Africa (Van Staden 2020). He was very critical of the deteriorating state of South Africa's transportation system, which effectively deprived China of opportunities to extract mineral resources from the mines (Fabricius 2020).

In 2018, the Chinese embassy condemned exiled Tibetan President, Lobsang Sangay's visit to South Africa and Pretoria's refusal to expel him. Lin Songtian reacted to what he referred to as a “betrayal of trust,” and thus,

It has sent a wrong political signal to the world community and has undermined the political mutual trust between China and South Africa. It runs against the common interest of SA-China relations, and will undoubtedly discourage Chinese investors’ confidence in South Africa, undermine SA's efforts for poverty reduction, and cause grave harm for the interest of South Africa and the South African people. (Fabricius 2020)

This validates the insinuation that, like other imperial powers, China's interest in Africa is purely economic. The timing of Lin's recall was of concern to foreign policy analysts and observers of South Africa–China relations. Different reasons have been cited for his departure, with some claiming that he was promoted and others of the view that Beijing recalled him temporarily as a protest against Pretoria's failure to post an ambassador to Beijing for more than a year. Another source maintained that it was related to his outburst against the United States following President Donald Trump's comments on the virus, while a few related it to the outbreak of the pandemic and the expatriation of South Africans from China (Fabricius 2020).

Due to South Africa's support for China following the outbreak of COVID-19, Chinese President, Xi Jinping promised to reciprocate by supporting South Africa's efforts to combat the pandemic (VOA 2020). He also enjoined Chinese migrants to support the country's efforts to counter the spread of the disease. Indeed, Beijing's COVID-19 diplomacy is visible in its relations with Pretoria, exemplified by the former's assistance to the latter in its efforts to combat the pandemic. On April 13, 2020, China dispatched a consignment of medical supplies to South Africa comprising 50,000 surgical masks, 11,000 N95 masks, 3,000 protective suits, 500 portable infrared thermometers, 11,000 pairs of surgical gloves, 3,000 goggles, and 11,000 pairs of medical shoe covers (South African Government News Agency 2020). Expressing his gratitude to China, then South African Minister of Health Zweli Mkhize noted that the donation would help to protect the country's health workers (South African Government News Agency 2020). In September 2020, the Chinese embassy in South Africa donated $59,700 as well as 50,000 surgical masks and 400 forehead thermometers (Xinhua 2020). Having received previous donations including 30,000 masks, 400 suits, and 500 thermometers from China in November 2020 (Defence Web 2020), the South African National Defence Force (SANDF) received 300,000 doses of the CoronaVac manufactured by Sinovac (Daily Maverick 2021). However, in light of the relative abundance of vaccines regarded as more effective such as Pfizer, Johnson & Johnson, and AstraZeneca, South Africa has been reluctant to administer Chinese vaccines and has donated its stockpile to other African states. Chinese companies have also come to the party. For example, Huawei Technologies C. Ltd. donated a million rands to South Africa's Department of Health and the Bank of China donated 100,000 face masks, 600 protective gowns, and five ventilators to the department. Like many African countries, especially in sub-Saharan Africa, South Africa relies heavily on China for the supply of personal protective equipment (PPE). In 2019, 18.3 percent of the country's imports of PPE came from China, with the figure increasing to 55.6 percent in 2020 (China Power 2021). These are clear indications of how Beijing has offered assistance to Pretoria in the fight against the pandemic.

However, South African citizens apparently expected much more, especially given the benefits enjoyed by Chinese investors in South Africa and the significant contributions from other sources. For instance, the Industrial Development Corporation (IDC) earmarked R3 billion for the procurement of essential medical kits. According to the South African President, the IDC “approved R130 million in funding and expects to approve a further R400 million in the coming week to companies who applied for funding under this special facility” (The Presidency 2020).

Following Xi Jinping's announcement on August 5, 2021, at the International Forum on COVID-19 Vaccine Cooperation that Beijing aims to produce two billion COVID-19 vaccine doses for the global market in 2021 and the pledge of $100 million to the COVID-19 Vaccine Global Access Facility (COVAX), Naledi Pandor South African Minister of International Relations and Cooperation commended China's efforts to fight the pandemic and urged other states to follow suit (Chinese Embassy in South Africa 2021). China has reiterated its commitment to assist South Africa and to ensure that Pretoria receives timeous delivery of Chinese vaccines.

The emergence of COVID-19 and its devastating effects on African states, economies, and societies did not spark hostility toward China on the continent. Despite the fact that the virus emerged from China, its nationals in Africa have not been subjected to any form of official or unofficial discrimination. However, after China declared itself free of the virus, it faced a diplomatic crisis on the continent due to its alleged COVID-19-related discrimination against African nationals (Marsh 2020). Given rising fears of imported and second-wave infections in China, foreign students of African descent—including those from South Africa—were subjected to forced COVID-19 testing and 14-day self-quarantine, with many evicted from their residences by landlords and rejected by hotels in parts of the country (Lindeque 2020). China's humiliation of and hostility to South Africans residing in Guangdong and Guangzhou became part of the public discourse in South Africa around mid-April 2020. According to the report, South Africans were locked inside and were only able to open their doors for food three times a day (Lindeque 2020).

The media in countries such as Uganda, Ghana, Kenya, and Nigeria also reported this alleged discrimination, prompting the chairperson of the AU Commission, Moussa Faki Mahamat to request an explanation from Beijing (Marsh 2020). Eventually, the state-owned *Global Times* responded to the continued diplomatic fallout and accused Western media of trying to damage China–Africa relations (Marsh 2020). While there might be some truth in this allegation, news of the discrimination, backed by audio and video clips was real and not provoked by the Western media. The outbreak of the virus in China, its handling of the disease and cases of racism and xenophobia against South Africans in China raise further questions on the future of South Africa–China relations.

## Conclusions

This article presented an overview of South Africa–China relations in the context of the COVID-19 pandemic. China's engagement with South Africa should be understood within the context of broad China–Africa relations. China seems less aggressive than the Western imperial powers in its dealing with African countries. Nevertheless, it may not be the “messiah” that will rescue Africa from its socio-economic and political oppression by global powers. While Beijing has maintained closer ties with South Africa since 1998 and since the country became a member of BRICS in 2010, South Africa has clearly benefited less in what many refer to as an asymmetric relationship. South Africa should utilize its status as the “gateway” to Africa to renegotiate its partnership with Beijing and demand more concessions in terms of Chinese investment and trade policy.

It is also imperative for the South African government to demand more support from China in the fight against COVID-19. In the initial stages, Lin Songtian projected China's response to COVID-19 and its spread to South Africa. While he was a popular figure in Beijing, Pretoria did not take kindly to his utterances. China's response to Africa's struggle against the pandemic has not demonstrated special treatment for South Africa. Based on Beijing's closer ties with the country, its membership of BRICS and other trade partnerships, it is understandable that South Africa expects more commitment from China.

Apart from Beijing's economic diplomacy with Pretoria, its propensity to “willingly” commit fully to the eradication of this health disaster in South Africa contradicts Beijing's character. This is due to the asymmetric relations between the two countries, putting South Africa at a disadvantage. While countries like the United States have castigated China for the outbreak and mishandling of the virus through the legal proceedings initiated by Missouri state against China (Harper 2020), South Africa should put more pressure on Beijing for “compensation” by significantly investing in the development of South African health infrastructure as the health sector has faced many challenges in the past few years. Sincere commitment to combating the spread of the disease is the minimal diplomatic intervention expected of a close ally. As it stands, Beijing's “coronavirus diplomacy” has rightly—drawn displeasure from a cross-section of South African society.
